# Anthropometric and nutritional analysis of Portuguese retail workers for ergonomic design applications: a cross-sectional study in northern Portugal

**DOI:** 10.3389/fpubh.2026.1822394

**Published:** 2026-09-17

**Authors:** Tânia T. Silva, Eduarda Dias, Inês Lapa, D-Filipa Ferreira, Paulo C. A. Filho, Ana Colim, Pedro Arezes, Paulo Carvalho, Matilde A. Rodrigues

**Affiliations:** 1 RISE-Health, Center for Translational Health and Medical Biotechnology Research (TBIO) ESS, Technical University of Porto, Porto, Portugal; 2 Department of Environmental Health, ESS, Technical University of Porto, Porto, Portugal; 3 ALGORITMI Research Center/LASI, University of Minho, Guimarães, Portugal

**Keywords:** anthropometry, equipment design, ergonomics, nutritional status, supermarkets

## Abstract

**Introduction:**

Anthropometric and nutritional status data are essential for optimizing working conditions, workstation design, and occupational health strategies, contributing to the prevention of work-related disorders and accidents. The retail sector is particularly relevant due to its diverse workforce and frequent exposure to prolonged standing, manual handling, and high physical demands.

**Methods:**

This cross-sectional study aimed to develop an anthropometric database and assess the nutritional status of workers from a Portuguese food retail company, providing evidence to support ergonomic workstation and task redesign, as well as initiatives to promote worker health. Data were collected from 155 supermarket workers in Northern Portugal (117 women and 38 men, aged 18–64 years). A total of 28 anthropometric measurements and 7 body composition parameters were assessed.

**Results:**

Men generally presented larger mean dimensions than women, except for hip breadth and seated elbow height. Men also showed a higher prevalence of overweight, while obesity was more frequent among women.

**Discussion:**

The findings highlight a high prevalence of excess weight and low physical activity levels, reinforcing the need to update anthropometric data and integrate inclusive ergonomic design and workplace wellness programs.

## 1 Introduction

Anthropometry involves the study of body characteristics such as body segments, lengths, circumferences, widths, heights, strength, and work capacity (1). It plays a significant role in the field of human sciences and health, as well as in the design of different kinds of products and spaces, such as clothing, tools, and work equipment (1). When properly applied to design and occupational health, anthropometry can enhance well-being, functional efficiency, usability, comfort, and health and safety (1, 2). On the other hand, Ergonomics (or human factors) is the scientific discipline concern with understanding how humans interact with various elements of a system (3), and applies theoretical knowledge, principles, data, and methods to design processes and enhance human well-being and optimize overall system performance. Ergonomists play a key role in designing and evaluating tasks, jobs, products, environments, and systems to ensure they align with human needs, abilities, and limitations (4). However, the use of up-to-date anthropometric data is crucial to this process (1, 2). When inadequate data are used, or ergonomic requirements are not considered, the resulting design fails to fit the majority of workers, inducing biomechanical stress on joints and muscles, elevating the risk of musculoskeletal disorders and occupational diseases (2), adversely affecting posture (5), and undermining physical well-being, cognitive performance, and work performance (6).

To guarantee its utility, anthropometric data must reflect the dimensions of the target population - those for whom the design is intended – which requires surveying workers’ body dimensions before implementing new products or equipment in their workplace (7). Gathering such data at a national scale can be time-consuming, expensive, and complex. As a result, there is increasing interest in collecting anthropometric data locally, particularly within specific industries (8). Anthropometric databases have therefore been developed worldwide to aid in the design of products and equipment suited to specific populations, demonstrating their value for working populations such as firefighters in the UK (9) and truck drivers in the US (10). However, there is a lack of updated anthropometric data for Portuguese workers, particularly in the retail sector, which limits effective ergonomic design and increases the risk of a mismatch between workers and their work environments (11, 12). This challenge is compounded by the need for universal design that ensures comfort for all individuals.

Beyond anthropometry, assessing workers’ nutritional status is also important because body composition influences not only general health but also physical capacity and biomechanical responses during occupational tasks. Therefore, evaluating indicators such as body mass index, body fat percentage, visceral fat, and muscle mass provides complementary information that can support ergonomic interventions and workplace design (13, 14). Excess body mass, particularly central adiposity, modifies body geometry and alters postural alignment, thereby increasing mechanical loading on the spine and lower-limb joints during standing, walking, lifting, and repetitive work tasks. In manual material handling, obesity may increase spinal compression forces and joint moments due to the greater mass that must be stabilized during movement. Furthermore, reduced relative muscle strength and mobility associated with excess adiposity may compromise movement efficiency, increase fatigue, and reduce the ability to adopt neutral postures. These biomechanical changes can contribute to a higher risk of work-related musculoskeletal disorders (WMSD), particularly in occupations involving repetitive movements, prolonged standing, awkward postures, and manual handling tasks.

The retail sector is one where specific anthropometric data are particularly relevant, given the notably high prevalence of WMSD reported by Kihlstedt and Hägg (15) and Skovlund et al. (16). The inappropriate design of workstations (e.g., cashiers station) and equipment (e.g., shelves) are frequently reported as important risk factors of WMSD in the retail sector and numerous studies over the years have explored its prevalence (12, 17–21), identifying contributing factors related to design, including scanner features (22–24) checkout dimensions, and other features that jeopardize cashier’s posture (15, 22, 25–28). The mismatch between workers’ requirements and the equipment may be exacerbated by the presence of customers. For example, checkout design can significantly influence customer behavior, potentially resulting in stress for cashiers and contributing to the onset of various health issues affecting the neck, shoulders, wrists, and hands (15).

It is therefore essential to assess comprehensively the anthropometric characteristics and nutritional status of workers in this sector, because both affect their health and well-being at work (13, 14). Nevertheless, the current literature highlights a notable lack of evidence on the nutritional status of workers in the retail sector, particularly in Portugal. Few studies have specifically addressed this issue, despite the fact that suboptimal nutrition not only affects physical health but also compromises work capacity and performance, and may lead to increased absenteeism, decreased productivity, and heightened susceptibility to work-related injuries and illnesses (29). Consequently, promoting healthy eating habits and providing access to nutritious food options in the workplace is essential for fostering a supportive and thriving work environment (30).

By collecting anthropometric measurements and assessing workers’ nutritional status and body composition, this study aims to develop a comprehensive anthropometric database for the Portuguese retail food sector to support the redesign of equipment, tools, and workstations to accommodate workers’ physical characteristics. The resulting data aim to improve ergonomic design, reduce health and safety risks, and support workplace adjustments. By integrating anthropometric and body composition data, this database contributes to the development of evidence-based ergonomic interventions, promotes safer and more inclusive work environments, and supports strategies to reduce WMSD and improve occupational health monitoring.

## 2 Materials and methods

### 2.1 Sample characterization

The research was conducted within the largest food retail chain in Portugal, at a supermarket in the northern Portugal. This store employs approximately 390 workers and is among the chain’s establishments with the largest workforce. Data collection took place between June and August 2023.

Before data collection, the research team contacted store management and presented the study objectives and data-collection procedures. All workers employed at the store were invited to participate to recruit as many as possible within the available time for data collection. This study applied no specific exclusion criteria. Participation was voluntary, and all invited workers agreed to participate; therefore, there were no refusals or non-participants.

During the data collection period, the store made 155 workers available to participate in the study. Consequently, the final sample comprised 155 workers from different supermarket sections: Bakery; Bazaar; Bazaar - flows; Bazaar - supply; Butchery; Cafeteria; Cashier station; Charcuterie; Cleaning supplies - flows; Customer Service; Food; Food Safety; Food Supply; Fresh; Fresh - supply; Fruits and vegetables; Grocery/drinks - flows; Non-food; Seafood; Reception; Sales - bazaar; Sales - food; Sales – textile; Takeaway and Textile. This sample accounted for 39.74% of the total supermarket workforce. The participation flow—from the total store workforce (approximately 390 employees), through universal invitation, to the 155 workers available for measurement during the data-collection period—is summarized above. The minimum required sample size was calculated using the standard formula for estimating a population proportion, *n*₀ = [*Z*^2^·*p*·(1 − *p*)] / *e*^2^, where *n*₀ denotes the uncorrected sample size, with a 95% confidence level (*Z* = 1.96), an estimated proportion of *p* = 0.2 (value adopted in the absence of a pre-established outcome-specific prevalence estimate for this population), and a margin of error of *e* = 0.05. This uncorrected estimate was then adjusted for the finite size of the source population (*N* ≈ 390 workers) using the finite population correction *n* = *n*₀ / [1 + (*n*₀ − 1)/*N*], yielding a minimum benchmark sample size of 151 participants. The final sample of 155 workers exceeds this benchmark. This calculation should be understood as a general feasibility benchmark rather than a formal justification of statistical adequacy for the study’s main outputs, which are descriptive anthropometric statistics and sex-specific percentile estimates; as discussed in Section 4.1, the smaller male subgroup (*n* = 38) means that extreme percentiles (P5/P95) for men should be interpreted with appropriate caution.

### 2.2 Study design

For this cross-sectional study, reported in accordance with the STROBE Statement guidelines (31), data collection was conducted by two researchers who served as evaluators and recorders. The protocol included measurements at various times of the day to capture different shifts.

Before data collection, each worker attended an introductory session about the study and had an opportunity to ask questions. Each participant then provided written informed consent in accordance with the guidelines of the Declaration of Helsinki, and the study was approved by the Ethics Committee of the School of Health of the Polytechnic Institute of Porto (approval number CE0061D) on 4 July 2023.

A self-reported questionnaire was administered to collect demographic data, including age, dominant hand, and level of physical activity. Physical activity was categorized based on weekly duration of activity (excluding occupational physical activity) into three levels: inactive (<30 min/week), moderately active (30–60 min/week), and active (>60 min/week). Additionally, measurements relevant to the body composition assessment were collected using a Tanita body composition scale (model BC-543; Japan; see Figure 1). The following body-composition parameters were measured: body mass, fat percentage, body water percentage, muscle mass, physical classification (body type), bone mass, and visceral fat index. These data also served to determine body mass index (BMI), a measure of nutritional status in adults (32). These parameters were used to classify nutritional status according to World Health Organization (WHO) BMI criteria^1^ and to characterize body composition for ergonomic design purposes. After these procedures, static anthropometric dimensions were collected.

**Figure 1:**
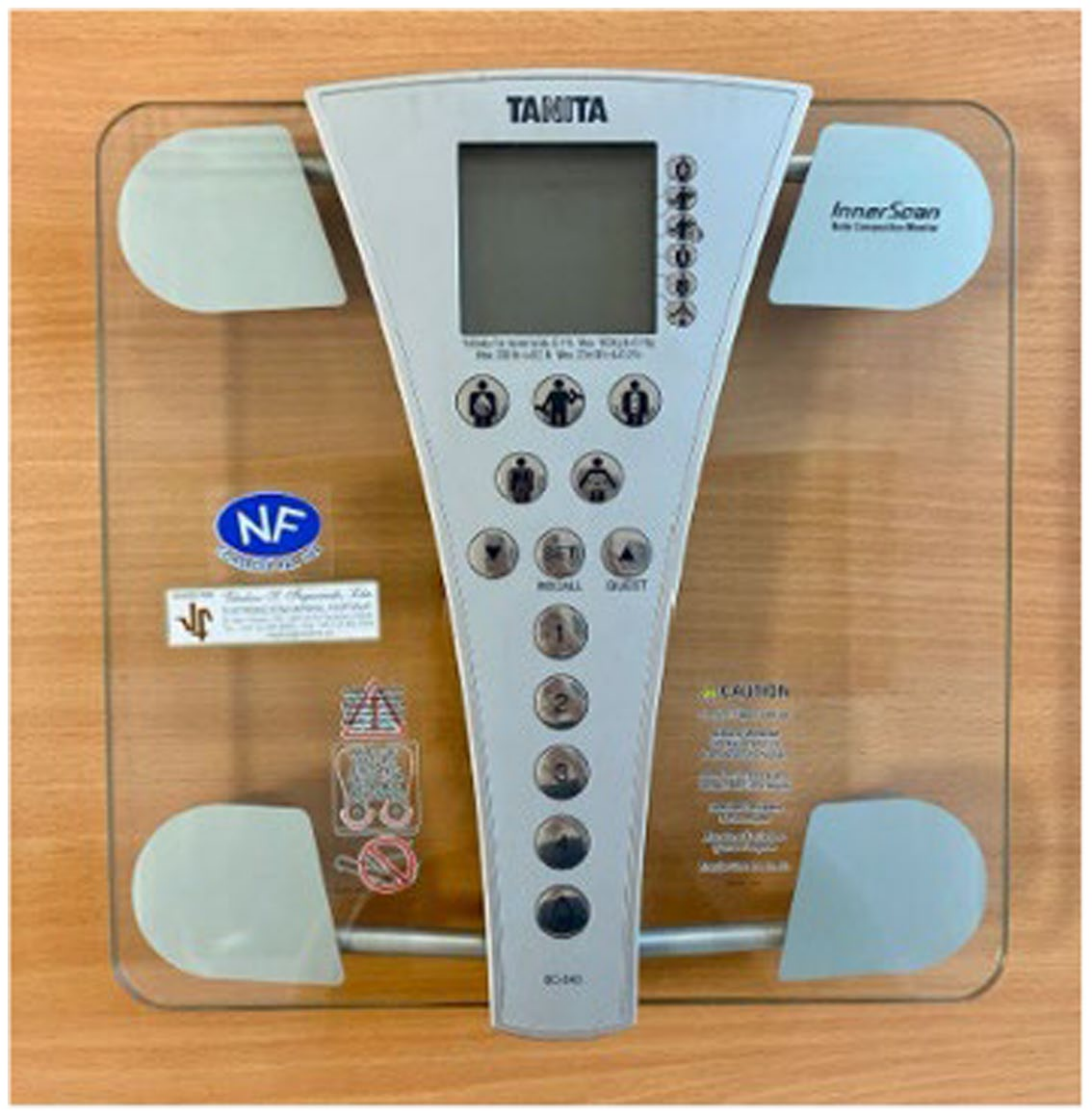
Tanita body composition scale.

### 2.3 Equipment and procedure for collection anthropometric dimensions

For this study, 28 static anthropometric dimensions relevant to workstation design in the food retail industry were measured for each participant. Of these dimensions, 7 were measured with the participant standing, while the remaining 21 were measured in a seated position. These dimensions were selected based on previous anthropometric studies (see (2, 8, 33)) and a prior analysis of the target equipment and workstation design requirements.

The anthropometric dimensions were obtained using the following equipment (see Figure 2): (a) a stationary anthropometer, (b) two dynamic anthropometers (one small and one large) (RealMet Institute, Barcelona, Spain), and (c) a height-adjustable stool with four different height settings (300, 350, 400, and 450 mm).

**Figure 2:**
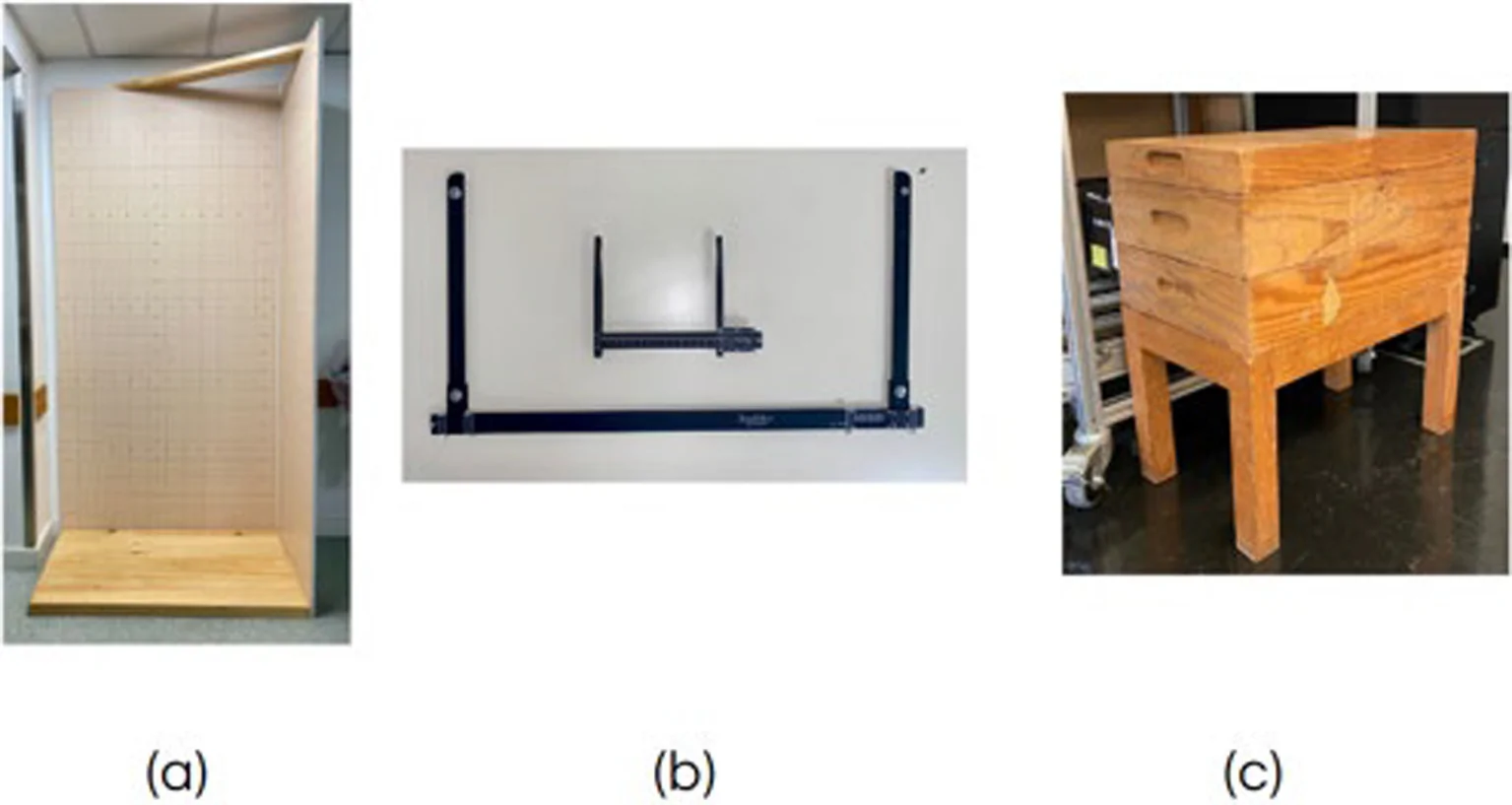
Equipment used for taking anthropometric measures: **(a)** stationary anthropometer; **(b)** dynamic anthropometers; **(c)** height-adjustable stool.

The dynamic and stationary anthropometers were used in conjunction with the height-adjustable stool to accurately measure dimensions while participants were seated. All equipment was calibrated in accordance with the relevant requirements; therefore, recalibration between measurements was unnecessary.

Anthropometric measurements followed ISO 7250-1 (34). All measurements were taken on the right side of the body, and the measurement definitions were based on Pheasant and Haslegrave (1) and ISO 7250-1 (34) (Table 1).

**Table 1:** Description of the measured anthropometric dimensions.

Equipment^a^	Dimension	Abbreviation	Definition^b^
Standing			
SA + SDA	Stature	Stature	Vertical distance between the floor and the top of the head measured with the subject erect and looking straight ahead (Frankfort plane)
SA + SDA	Vertical grip reach	VGR	Distance from the floor to the center of a cylindrical rod grasped in the palm of the hand, with the arm raised vertically above the head in an easy reach (without excessive stretch)
SA + SDA	Eye height	EyeH	Vertical distance from the floor to the outer corner of the eye
SA + SDA	Shoulder height	ShoulderH	Vertical distance from the floor to the acromion
SA + SDA	Elbow height	ElbowH	Vertical distance from the floor to the lowest bony point of the bent elbow
SA + SDA	Knuckle height	KnuckleH	Vertical distance from the floor to metacarpal III
SA + SDA	Forward grip reach	FGR	Horizontal distance from a vertical surface to the grip axis of the hand while the subject leans both shoulder blades against the vertical surface
Sitting			
SA + SDA + AS	Height (Sitting)	SittingH	Vertical distance between the subject’s seated surface and the top of the head measured with the subject erect and looking straight ahead (Frankfort plane)
SA + SDA + AS	Vertical grip reach sitting	SittingVGR	Distance from the seat surface to the center of a cylindrical rod grasped in the palm of the hand, with the arm raised vertically above the head in an easy reach (without excessive stretch)
SA + SDA + AS	Eye height sitting	EyeHS	Vertical distance between the sitting surface to the inner canthus (corner) of the eye (head in Frankfort plane)
SA + SDA + AS	Shoulder height sitting	ShoulderHS	Vertical distance from subject’s seated surface to the acromion
SA + SDA + AS	Elbow height sitting	ElbowHS	Taken with a 90° of elbow flexion. Vertical distance from the lowest point of the elbow (olecranon) to the subject’s seated surface
SA + SDA + AS	Buttock-knee length	BKL	Horizontal distance from the foremost point of the kneecap to the rearmost point of the buttock
SA + SDA + AS	Knee height	KneeH	Vertical distance from the floor or footrest to the highest point of the superior border of the patella
SA + SDA + AS	Buttock-popliteal length	BPL	Horizontal distance from the popliteal surface to the rearmost point of the buttock
SA + SDA + AS	Popliteal height	PoplitealH	Vertical distance from the floor or footrest to the posterior surface of the knee (popliteal surface)
LDA + AS	Thigh clearance	ThighC	Vertical distance from the highest uncompressed point of the thigh to the subject’s seated surface
SA + SDA + AS	Elbow grip length	EGL	Horizontal distance from back of the upper arm (at the elbow) to grip axis, with elbow bent at right angles
LDA + AS	Abdominal depth	Abdominal	Maximum horizontal distance from the vertical reference plane to the front of the abdomen in the standard sitting position
LDA + AS	Elbow to elbow breadth	ETEB	Maximum horizontal breadth across the elbows
LDA + AS	Shoulder breadth (bideltoid)	SBD	Distance across the maximum lateral protrusions of the right and left deltoid muscles
LDA + AS	Shoulder breadth (biacromial)	SBA	Distance between the outermost bony points on the top of each shoulder
LDA + AS	Hip breadth	HipB	Horizontal distance measured at the widest point of the hip in the sitting position
LDA + AS	Subscapular height	SSH	Vertical distance from the lowest point (inferior angle) of the scapula to the subject’s seated surface
LDA + AS	Hand length	HandL	Perpendicular distance from a line drawn between the styloid processes to the tip of the middle finger
SDA + AS	Hand breadth	HandB	Projected distance from the radial side of the second metacarpal to the ulnar side of the fifth metacarpal at the level of the metacarpophalangeal joints
LDA + AS	Foot breadth	FootB	Maximum distance between medial and lateral surfaces of the foot perpendicular to the longitudinal axis of the foot
LDA + AS	Foot length	FootL	Maximum distance from rear of the heel to tip of the longest (first or second) toe, measured parallel to the longitudinal axis of the foot
Scale	Body mass	BM	Total mass of an individual’s body, expressed in kilograms (kg).
Calculated			
Calculated through a formula	Body mass index	BMI	Calculated using the formula: BMI (kg/m^2^) = body mass (kg)/stature (m^2^)

a Stationary anthropometer (SA), Large Dynamic anthropometer (LDA), Small Dynamic anthropometer (SDA), Adjustable stool (AS).

b Sources: ISO 7250-1:2017 and Pheasant and Haslegrave (1).

The measurement procedure began with the participant standing at the stationary anthropometer, where the standing dimensions were collected. Participants were then seated on the height-adjustable stool, positioned within the stationary anthropometer, where the seated dimensions were measured with the dynamic anthropometers. For measurements that required a seated position, the stool’s height was adjusted to ensure a 90-degree knee angle for each worker. Measurements for each participant typically took approximately 15 min.

### 2.4 Pilot study: intra- and inter-measurer reliability

The anthropometric measurements were collected by two evaluators who had prior knowledge of ergonomics and anthropometry. Additional evaluators could not be recruited; therefore, the study was conducted with two evaluators. Before data collection, both evaluators underwent standardized training on equipment handling, anthropometric measurement procedures, and the definitions of the measurements.

To validate the measurement procedure and assess the accuracy of the data acquisition, a pilot study was conducted with 22 participants representing a diverse range of anthropometric dimensions [i.e., the fifth (P5), fiftieth (P50), and ninety-fifth (P95) percentiles] and all genders. These participants were not part of the anthropometric study sample.

Each of the 28 static anthropometric measurements was taken twice for each participant by each evaluator. For data analysis, the intraclass correlation coefficient (ICC) was determined. Assessing inter- and intra-evaluator reliability can improve confidence in measurement consistency (35). Inter-evaluator reliability assesses the level of consistency or agreement in readings between two evaluators for the same subjects. Intra-evaluator reliability assesses the consistency or agreement in readings by the same evaluator for the same subjects at two distinct time points (36).

We used the ICC to assess consistency both within evaluators (intra-evaluator reliability) and between evaluators (inter-evaluator reliability). The ICC, ranging from 0.00 to 1.00, is interpreted as follows: ICC ≤ 0.50 indicates weak reliability, 0.50 < ICC < 0.75 suggests moderate reliability, ICC ≥ 0.75 indicates strong reliability, and ICC ≥ 0.90 indicates excellent reliability (37).

In the current study, Intra-evaluator ICC values for evaluator 1 ranged from 0.968 to 0.999, while those for evaluator 2 ranged from 0.989 to 1.00. The inter-evaluator ICC values ranged between 0.905 and 0.998, indicating excellent reliability.

### 2.5 Data analysis

Because no discrepancies were found between assessors and the pilot study demonstrated excellent intra- and inter-rater reliability, each participant was assessed by only one assessor in the main study, rather than independently by both. No missing data were recorded for any of the anthropometric, body composition, or demographic variables analyzed, as all 155 participants completed the full measurement protocol.

All collected data were stored in Microsoft® Excel (2016) software. After validation and error checking, the data were analyzed using Microsoft Excel 2016 and IBM SPSS Statistics 28 (2021). Descriptive statistics, including means, standard deviations, and the 5th, 50th, and 95th percentiles, were calculated using these software packages. The results were presented in anthropometric tables, and inferential tests were also conducted. The normality of the distributions was verified using the Kolmogorov–Smirnov test. Spearman’s rank correlation coefficient was used to examine correlations among the following variables: age, gender, level of physical activity, height, body mass, percentage of body fat, percentage of total body water, muscle mass, body type, bone mass, visceral fat index, and BMI. The significance level was set at *p* < 0.05. The normality of BMI within each physical activity group was assessed using the Kolmogorov–Smirnov test, and homogeneity of variances was evaluated using Levene’s test. As both assumptions were met, a one-way analysis of variance (ANOVA) was performed to compare mean BMI across the three physical activity levels. Partial eta squared (η^2^p) was calculated as a measure of effect size. *Post hoc* pairwise comparisons would be performed only if the overall ANOVA was statistically significant. For the principal correlation and ANOVA estimates highlighted in the Results, 95% confidence intervals (CIs) were additionally computed: for Spearman correlation coefficients, using Fisher’s z transformation; for partial eta squared, using the noncentral F distribution (Steiger’s method).

## 3 Results

### 3.1 Sample characterization

Participants in the study were selected through convenience sampling, considering both their availability and willingness to participate. The sample represented 39.74% of the total workforce. The gender distribution in the study was similar to that of the company, with females accounting for 75.5% in the study (73.33% in the supermarket) and males representing 24.5% (26.67% in the supermarket). The age of participants ranged from 18 to 64 years.

Figure 3 presents the sample distribution according to age category and gender. The majority of participants were female (75.5%). The mean age was 45 (SD = 14.0) years for women and 38 (SD = 14.7) years for men. A total of 56.13% of participants were classified as middle-aged according to WHO criteria (38). Regarding physical activity (excluding occupational activity), 66% of participants were classified as inactive, 14% as moderately active, and 20% as active (Figure 4). Concerning hand dominance, 92.26% of participants were right-handed, 6.45% left-handed, and 1.29% ambidextrous. The remaining Tanita-derived parameters (body fat percentage, total body water, bone mass, and visceral fat index) are presented in Table 2 (39). Table 3 presents body composition parameters and BMI values for females and males, including mean, standard deviation, and percentiles (P5, P50, P95). Based on the Tanita body composition classification system, participants were categorized into body types as shown in Table 3 (39).

**Figure 3:**
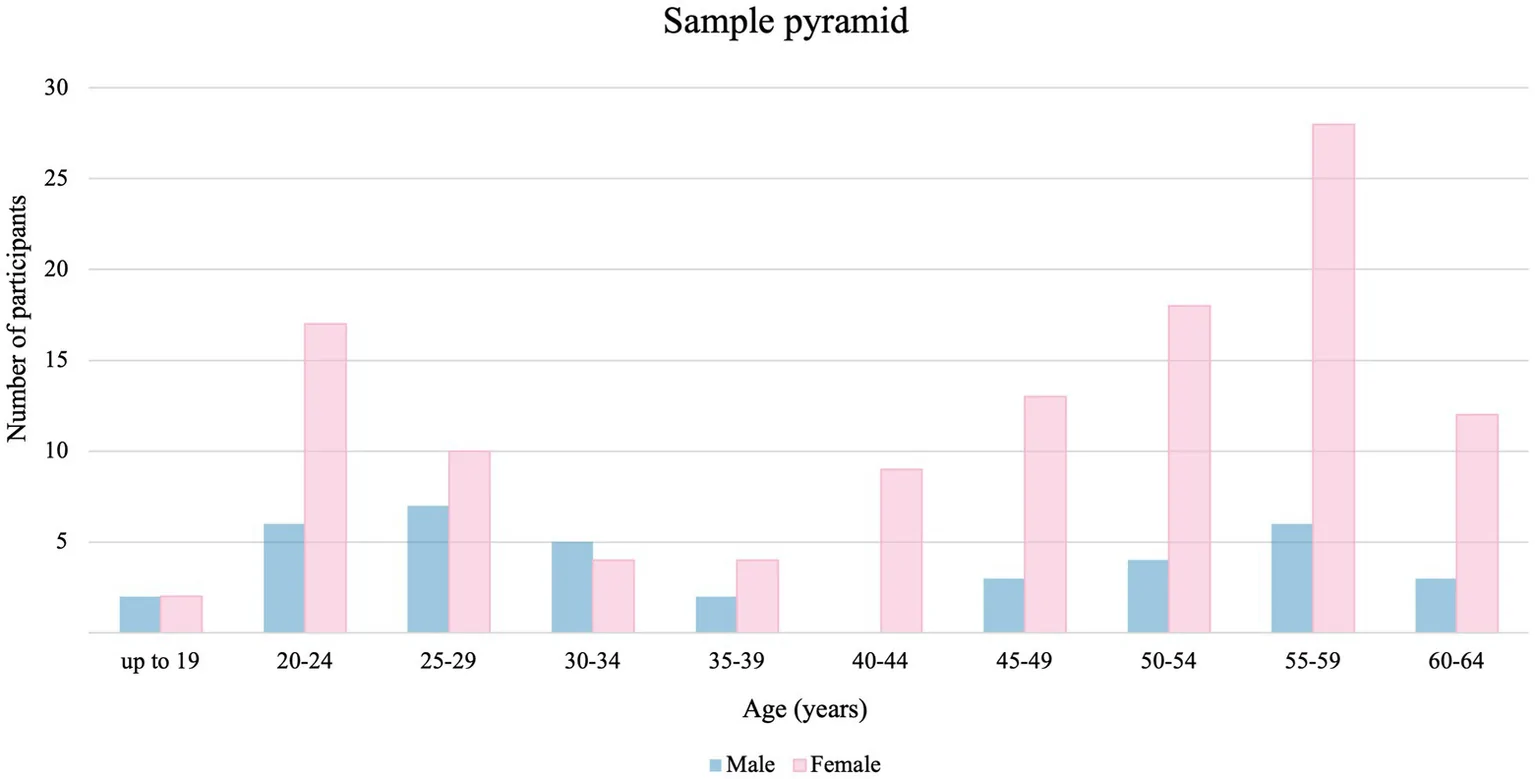
Sample distribution by age and gender.

**Figure 4:**
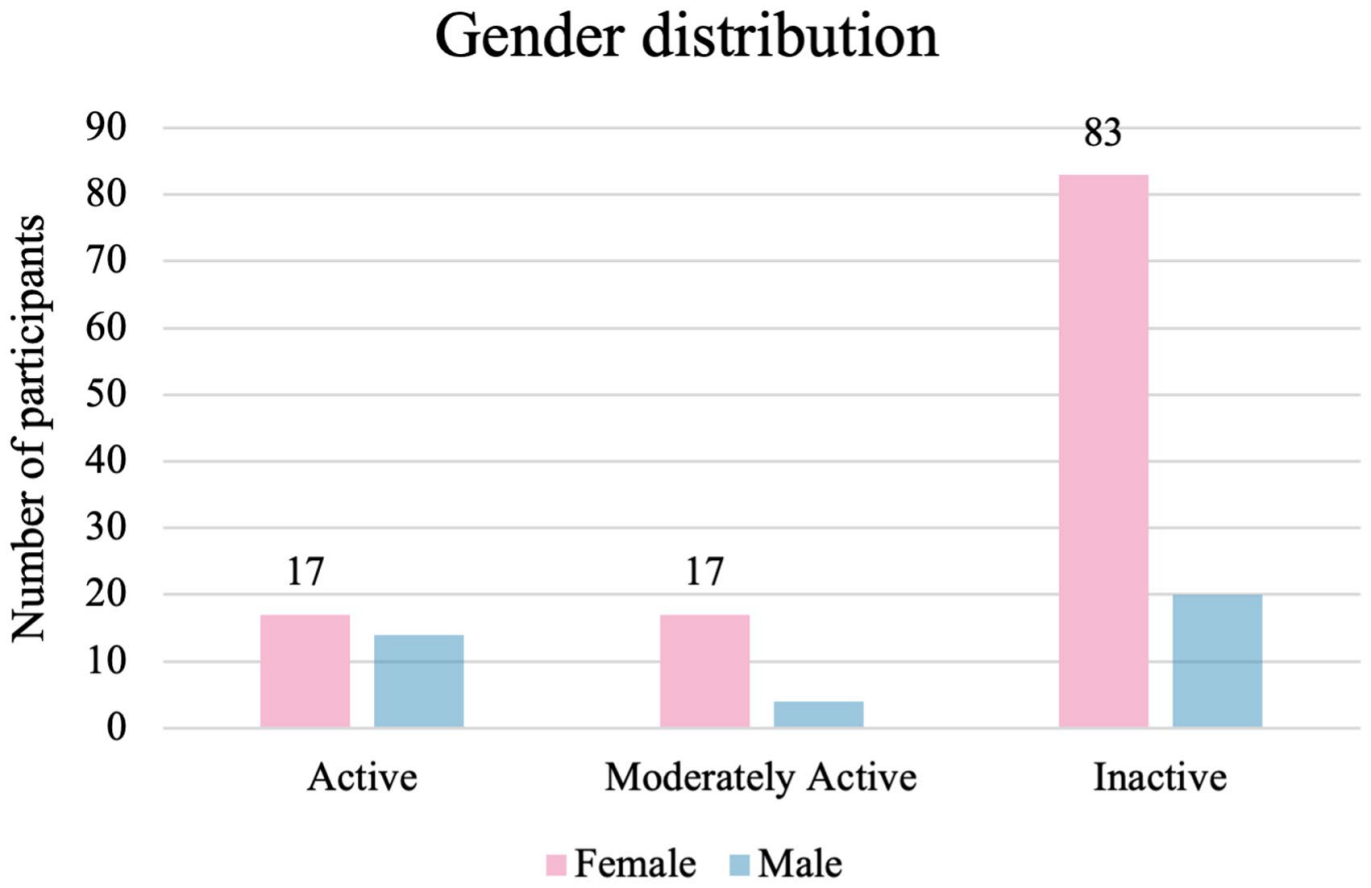
Level of physical activity by gender.

**Table 2:** Recommended values for the body composition parameters evaluated using the Tanita scale.

Parameters	Female			Male		
Body fat (%)	20–39 years	40–59 years	60–79 years	20–39 years	40–59 years	60–79 years
	21–33%	23–34%	24–36.5%	7–20%	10.5–22%	11.5–25%
Total body water (%)	45–60%			50–65%		
Bone Mass (kg)	less than 50 kg	50 kg – 75 kg	over 76 kg	less than 65 kg	65 kg – 95 kg	over 95 kg
	1.95	2.40	2.95	2.65	3.29	3.69
Visceral fat index	1–12 (healthy level)					

Source: Tanita, (39).

**Table 3:** Body composition parameters gathered in this study, along with the calculated BMI.

Body composition parameters and BMI*	Female					Male				
	Average	SD	P5	P50	P95	Average	SD	P5	P50	P95
BM (kg)	69.6	15.6	49.1	68.8	94.0	78.7	13.1	57.3	76.5	99.3
BFP (%)	34.7	8.2	20.4	35.9	45.6	22.5	7.6	10.0	23.3	32.8
TBW (%)	45.7	5.2	39.2	45.3	55.2	54.8	5.3	48.8	53.7	64.6
MM (kg)	42.1	4.5	36.4	42.0	48.4	57.4	7.3	47.8	55.6	68.6
BType (1–9)	3.7	1.6	2.0	3.0	7.3	3.1	1.4	2.0	2.0	5.0
BoneM (kg)	2.3	0.2	1.9	2.2	2.6	3.0	0.3	2.6	2.9	3.5
VF	7.1	3.3	1.0	7.0	13.0	8.5	5.3	1.0	8.5	15.2
BMI (kg/m^2^)	27.7	5.6	19.6	27.1	36.6	26.5	3.9	19.2	27.5	31.7

*Body mass (BM), body fat percentage (BFP), total body water (TBW), muscle mass (MM), body type (BType), bone mass (BoneM), visceral fat index (VF), body mass index (BMI).

### 3.2 Anthropometric dimensions

Table 4 presents the collected static anthropometric dimensions for both females and males. These data include means and standard deviations, as well as the fifth percentile (P5), fiftieth percentile (P50), and ninety-fifth percentile (P95).

**Table 4:** Anthropometric data gathered in this study.

Anthropometric dimensions	Female					Male				
	Average	SD	P5	P50	P95	Average	SD	P5	P50	P95
1 HandL (mm)	171	8	160	171	183	187	9	169	189	199
2 HandB (mm)	77	4	71	77	83	86	5	80	86	94
3 FootL (mm)	237	12	221	236	259	260	13	241	263	277
4 FootB (mm)	93	6	85	92	102	99	6	90	99	110
5 Stature (mm)	1,582	61	1,499	1,580	1,676	1725	80	1,616	1725	1863
6 VGR (mm)	1869	92	1716	1863	2005	2080	98	1942	2080	2,258
7 EyeH (mm)	1,481	61	1,396	1,480	1,587	1,621	79	1,504	1,621	1760
8 ShoulderH (mm)	1,293	55	1,207	1,289	1,382	1,418	70	1,308	1,415	1,552
9 ElbowH (mm)	990	43	934	987	1,065	1,075	51	997	1,077	1,182
10 KnuckleH (mm)	653	32	598	654	701	707	42	655	707	782
11 FGR (mm)	676	40	609	675	738	732	45	659	733	804
12 SittingH (mm)	852	33	798	853	904	909	39	849	910	970
13 SittingVGR (mm)	1,133	79	1,015	1,140	1,239	1,242	98	1,138	1,252	1,344
14 EyeHS (mm)	745	41	684	748	801	798	50	748	805	861
15 ShoulderHS (mm)	564	31	513	562	611	592	65	548	601	661
16 ElbowHS (mm)	263	30	222	262	315	263	26	221	265	304
17 EGL (mm)	329	39	294	322	360	368	43	323	363	422
18 BKL (mm)	579	34	531	578	628	606	38	546	610	663
19 KneeH (mm)	486	26	451	485	524	533	33	492	530	583
20 BPL (mm)	490	32	439	489	533	505	37	446	503	566
21 PoplitealH (mm)	381	24	350	380	417	429	31	385	427	501
22 Abdominal (mm)	223	43	166	216	301	237	39	191	238	311
23 ETEB (mm)	453	54	371	458	616	471	41	403	475	536
24 SBD (mm)	430	38	373	427	498	471	31	424	468	518
25 SBA (mm)	362	23	329	361	404	394	20	365	393	427
26 HipB (mm)	402	40	346	399	459	370	29	323	370	418
27 SSH (mm)	432	26	388	435	470	453	26	410	453	493
28 ThighC (mm)	151	25	114	149	193	151	18	128	146	185

*Hand length (HandL), hand breadth (HandB), foot length (FootL), foot breadth (FootB), stature (Stature), vertical grip reach (VGR), eye height (EyeH), shoulder height (ShoulderH), elbow height (ElbowH), knuckle height (KnuckleH), forward grip reach (FGR), sitting height (SittingH), vertical grip reach sitting (SittingVGR), eye height sitting (EyeHS), shoulder height sitting (ShoulderHS), elbow height sitting (ElbowHS), elbow grip length (EGL), buttock-knee length (BKL), knee height (KneeH), buttock-popliteal length (BPL), popliteal height (PoplitealH), abdominal depth (Abdominal), elbow to elbow breadth (ETEB), shoulder breadth bideltoid (SBD), shoulder breadth biacromial (SBA), hip breadth (HipB), subscapular height (SSH), thigh clearance (ThighC).

The data show that the average height (stature) and weight (BM) in the sample were, respectively, 1.72 m and 78.7 kg for men, and 1.58 m and 69.6 kg for women. The mean BMI was 27.7 kg/m^2^, for women and 26.5 kg/m^2^. These values placed all genders in the pre-obesity range (see Table 5), which shows the value ranges for BMI (32). The data also indicate that, except for hip breadth and elbow height sitting, men exhibited greater average body segmental dimensions than women.

**Table 5:** Distribution of the nutritional status of participants by gender based on World Health Organization, 2010.

BMI	Nutritional status	Male	Female
Below 18.5	Underweight	0%	2.56%
18.5–24.9	Normal weight	31.58%	30.77%
25.0–29.9	Pre-obesity	55.26%	33.33%
30.0–34.9	Obesity class I	10.53%	24.79%
35.0–39.9	Obesity class II	2.63%	5.98%
Above 40	Obesity class III	0%	2.56%

Table 5 presents the nutritional status of participants by gender. Among male participants, 31.58% were categorized as having a normal weight, 55.26% were classified as overweight, 10.53% fell into the category of obesity class I, and 2.63% were identified with obesity class II. Among female participants, 2.56% were underweight, 30.77% had normal weight, 33.33% were overweight, 24.79% had obesity class I, 5.98% had obesity class II and 2.56% had obesity class III.

### 3.3 Correlations between anthropometric measures and demographic factors

Correlation analysis results are presented in Table 6. Significant correlations (*p* < 0.01) were observed between age and body fat (*r* = 0.250, 95% CI [0.096, 0.392]), and age and visceral fat index (*r* = 0.539, 95% CI [0.417, 0.642]). Gender showed significant correlations with height (*r* = 0.637, 95% CI [0.533, 0.722], *p* < 0.01) and muscle mass (*r* = 0.712, 95% CI [0.624, 0.782], *p* < 0.01). Physical activity level was significantly correlated with height (*r* = 0.220, 95% CI [0.065, 0.365], *p* < 0.01). Gender also showed a significant positive correlation with bone mass (*r* = 0.711, 95% CI [0.623, 0.781], *p* < 0.01). An ANOVA comparing BMI across physical activity levels revealed no statistically significant differences, *F*(2, 152) = 1.84, *p* = 0.162, η^2^p = 0.024, 95% CI [0.000, 0.081]. Descriptively, inactive and moderately active participants had a mean BMI of 27.8, whereas active participants had a mean BMI of 25.8.

**Table 6:** Spearman correlations between the main variables.

Variable	1	2	3	4	5	6	7	8	9	10	11	12
(1) Age		−0.170^*^	−0.098	−0.306^**^	−0.067	0.250^**^	−0.304^**^	−0.275^**^	−0.147	−0.259^**^	0.539^**^	0.112
(2) Gender	−0.170^*^		0.198^*^	0.637^**^	0.292^**^	−0.554^**^	0.601^**^	0.712^**^	−0.189^*^	0.711^**^	0.129	−0.075
(3) Level of physical activity	−0.098	0.198^*^		0.220^**^	0.036	−0.156	0.150	0.165^*^	−0.001	0.167^*^	−0.157	−0.131
(4) Height	−0.306^**^	0.637^**^	0.220^**^		0.362^**^	−0.346^**^	0.388^**^	0.684^**^	−0.221^**^	0.684^**^	−0.022	−0.149
(5) Body mass (kg)	−0.067	0.292^**^	0.036	0.362^**^		0.533^**^	−0.471^**^	0.789^**^	−0.616^**^	0.786^**^	0.687^**^	0.840^**^
(6) Body fat (%)	0.250^**^	−0.554^**^	−0.156	−0.346^**^	0.533^**^		−0.989^**^	−0.009	−0.432^**^	−0.009	0.585^**^	0.796^**^
(7) Total body water (%)	−0.304^**^	0.601^**^	0.150	0.388^**^	−0.471^**^	−0.989^**^		0.090	0.382^**^	0.088	−0.555^**^	−0.747^**^
(8) Muscle mass (kg)	−0.275^**^	0.712^**^	0.165^*^	0.684^**^	0.789^**^	−0.009	0.090		−0.424^**^	0.994^**^	0.391^**^	0.440^**^
(9) Body type	−0.147	−0.189^*^	−0.001	−0.221^**^	−0.616^**^	−0.432^**^	0.382^**^	−0.424^**^		−0.423^**^	−0.580^**^	−0.562^**^
(10) Bone mass (kg)	−0.259^**^	0.711^**^	0.167^*^	0.684^**^	0.786^**^	−0.009	0.088	0.994^**^	−0.423^**^		0.398^**^	0.438^**^
(11) Visceral fat index	0.539^**^	0.129	−0.157	−0.022	0.687^**^	0.585^**^	−0.555^**^	0.391^**^	−0.580^**^	0.398^**^		0.770^**^
(12) BMI	0.112	−0.075	−0.131	−0.149	0.840^**^	0.796^**^	−0.747^**^	0.440^**^	−0.562^**^	0.438^**^	0.770^**^	

** *p* < 0.01.

* *p* < 0.05.

## 4 Discussion

Overall, men generally had larger mean body-segmental dimensions than women, with the exception of hip breadth and elbow height sitting (2, 33, 40). These findings are consistent with those reported by Barroso et al. (8) and Filho et al. (33), who used the same methodology. It should be noted, however, that Barroso et al. (8) did not provide age-stratified data, which limits the precision of direct comparisons with the current sample. Compared to these earlier studies of Portuguese workers (Table 7), the present sample shows a slightly higher body mass in both sexes and a marginal increase in stature, consistent with a secular trend previously noted (33, 41). The retail sample exhibited slightly shorter stature and higher body weight than the manufacturing workers studied by Filho et al. (33), though differences were smaller than those observed relative to the older dataset reported by Barroso et al. (8).

**Table 7:** Sample population characteristics and BMI comparison across Barroso et al. (8), Filho et al. (33), and the current study (body mass in kg, stature in mm, BMI in kg/m^2^).

Sample characteristics		Barroso et al. (8)	Filho et al. (33)	Current study
Gender	Male	55.2%	49.3%	24.5%
	Female	44.8%	50.7%	75.5%
Age	Less than 20			
	Male	10.2%	0.6%	5.27%
	Female	8.0%	0.0%	1.71%
	20–50			
	Male	81.9%	86.4%	60.52%
	Female	78.5%	62.1%	48.72%
	More than 50			
	Male	11.4%	13.0%	34.21%
	Female	10.0%	37.9%	49.57%
BM (kg)	Male	74.0	77.9	78.7
	Female	64	63.7	69.6
Stature (mm)	Male	1690.0	1740.7	1725.0
	Female	1565.0	1594.7	1582.0
BMI (kg/m^2^)	Male	25.9	25.7	26.5
	Female	26.1	25.2	27.7

The anthropometric percentiles provide practical information for workstation and equipment design. In particular, P5 and P95 values can be used as reference limits when considering the accommodation of workers across a broad range of body dimensions, depending on the specific design requirement. As illustrated in Figure 5, vertical grip reach, shoulder height, and knee height are relevant for shelving design and for defining accessible working zones, while forward grip reach and elbow height can inform checkout counter dimensions and reach distances. For seating, popliteal height, buttock-popliteal length, and hip breadth can guide seat height, depth, and width, supporting appropriate fit and postural comfort. These applications illustrate how the present anthropometric database can support more inclusive ergonomic design in food retail workplaces.

**Figure 5:**
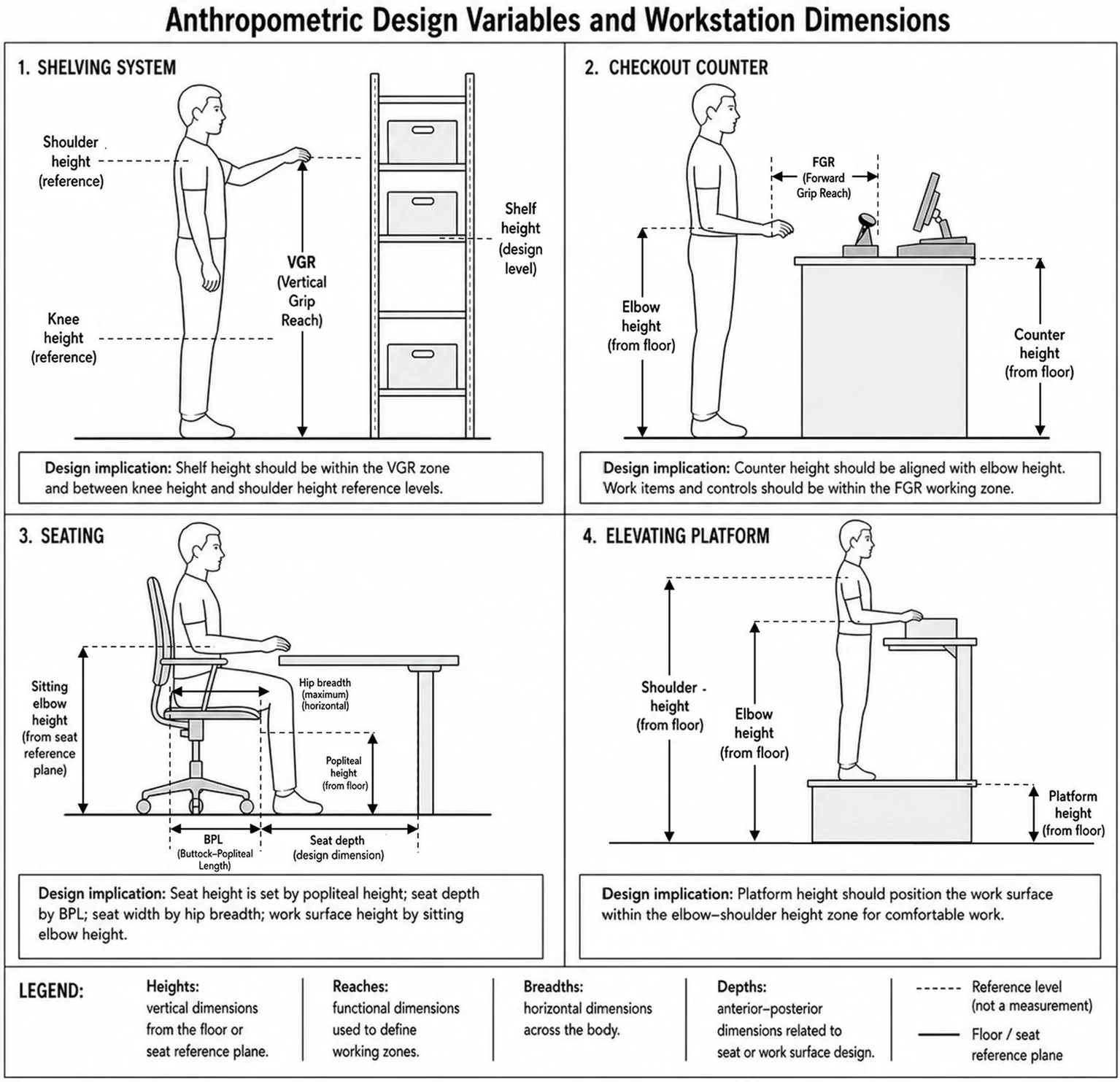
Anthropometric variables in workstation design.

The elevated BMI observed in this population is relevant from an ergonomic standpoint, as higher BMI combined with poorly designed workstations can exacerbate physical demands and increase musculoskeletal disorder risk (42, 43). Therefore, anthropometric and body composition information may be considered when developing ergonomic interventions aimed at reducing physical strain and potentially mitigating WMSD risk. However, these implications should be interpreted cautiously, as the present cross-sectional study did not directly assess biomechanical loading or musculoskeletal outcomes. Although the descriptive BMI was lower among active participants than among inactive or moderately active participants, the ANOVA showed no statistically significant differences in BMI across physical activity groups (*F*(2, 152) = 1.84, *p* = 0.162, η^2^p = 0.024). Thus, within this sample, there was insufficient statistical evidence of differences in BMI according to physical activity level. The result should therefore not be interpreted as evidence that physical activity and BMI are unrelated.

The predominance of women in the sample reflects the feminisation of retail employment, particularly in roles such as cashier and customer service positions, which may contribute to the higher BMI and lower physical activity levels observed among female workers (44–47). Overall, participants reported low levels of leisure-time physical activity, likely due to the demanding and irregular nature of food retail work (16). The associations identified between age, gender, physical activity, height, and body composition highlight the combined influence of biological, behavioral, and occupational factors on workers’ anthropometric profiles. For example, the identical mean thigh-clearance value of 151 mm observed for all genders, together with the gender-specific differences in seated and standing dimensions, have direct implications for workstation design and equipment specification in this sector. The positive correlation between height and physical activity may reflect cohort or socioeconomic differences, while the association between sex and bone mass is consistent with well-established differences in lean mass and body composition between males and females (48). Furthermore, higher per capita incomes, improved sanitation in housing and living conditions, enhanced health and nutrition education, and more robust social services and healthcare systems are key determining factors linked to increasing height (49).

The elevated prevalence of overweight and obesity observed in this sample is consistent with national trends reported by Camões et al. (50), who identified higher incidence rates of central obesity among women and older, less-educated individuals. These findings reinforce the need for workplace health interventions targeting this population. Inclusive wellness programs focused on physical activity and healthy behaviors have been shown to improve worker well-being and productivity across diverse body sizes (51). Employers are encouraged to implement tailored, evidence-based initiatives that account for participant characteristics and organizational constraints, given the heterogeneity in findings across intervention studies (52).

More than half of the adult population and over 30 percent of children aged 7 to 9 years have an unhealthy weight. Of particular concern is the rising prevalence of overweight and obesity over time. When compared to the neighboring Iberian country of Spain, the prevalence of obesity in Portugal does not differ significantly. Indeed, this mirrors the situation in most developed nations where overweight and obesity rates are high. This epidemiological context underscores the pressing need for more effective preventive strategies, with particular emphasis on targeted populations such as children and adolescents (53).

In the retail sector, the need for ergonomic improvements in workstations is evident, as poor workstation design significantly increases the risk of developing WMSD (18). Furthermore, workers’ health conditions can significantly affect job performance and productivity. Poor health may lead to reduced efficiency, increased absenteeism, and a greater likelihood of errors or accidents, which can, in turn, negatively affect work quality and workplace dynamics (54). To address this issue, employers should implement ergonomic and health interventions that can not only enhance workers’ health, safety, and productivity but also contribute to their overall well-being.

The anthropometric dataset generated in this study provides a direct evidence base for ergonomic design in the Portuguese retail sector. The measured dimensions can support the adaptation of shelving, checkout counters, seating, and picking stations to better accommodate the physical diversity of workers. For example, the implementation of elevating platforms at picking stations, informed by elbow and shoulder height data, can reduce excessive bending and overextension. These tailored solutions not only reduce the risk of musculoskeletal disorders and injuries but also improve comfort and productivity, benefiting both employees and employers (43).

### 4.1 Limitations

Despite the importance of the findings, a few limitations should be acknowledged. The relatively small convenience sample (*n* = 155) was drawn from a single food retail company operating in Portugal, which may limit representativeness and generalisability to other retail contexts or national populations. Although the gender distribution reflected that of the company, the lower representation of male participants may have limited the robustness of the sex-specific percentile estimates, particularly at the extremes of the distribution relevant for inclusive ergonomic design. In addition, body composition was assessed using bioelectrical impedance analysis (BIA), which is a practical method but provides lower accuracy than reference techniques. Self-reported physical activity data are subject to recall bias. Moreover, 95% confidence intervals are reported in Section 3.3 for the principal correlation and ANOVA estimates highlighted in the Results; confidence intervals were not computed for the remaining pairwise correlations in Table 6, for which only point estimates and significance levels are presented, consistent with the primarily exploratory purpose of that analysis. Furthermore, the cross-sectional design precludes causal inferences regarding the relationship between anthropometric characteristics and work-related health outcomes. In particular, the observed association between physical activity level and BMI should not be interpreted as causal, as the cross-sectional design cannot establish the direction of this relationship. The height-adjustable stool used for seated measurements offered only four fixed height settings (300, 350, 400, and 450 mm), which may not have allowed an exact 90-degree knee angle to be achieved for participants at the extremes of the height distribution, potentially introducing small measurement inaccuracies for these individuals. Moreover, dynamic anthropometric measurements were not performed, which may limit the applicability of the findings to tasks involving movement and changing postures. Nonetheless, the findings are consistent with those reported in similar studies. Future research should include longitudinal designs and broaden the sample to encompass a wider range of retail subsectors and regional populations.

## 5 Conclusion

This study provides a comprehensive anthropometric profile of Portuguese food retail workers, characterized by a predominantly female, middle-aged workforce with elevated BMI values and low leisure-time physical activity. The observed differences relative to earlier Portuguese datasets reflect secular trends in body weight and underscore the need for updated ergonomic standards tailored to this occupational group. These findings should be interpreted within the context of the study and should not be considered representative of the general Portuguese population. Instead, they provide occupation-specific anthropometric evidence that helps address the current lack of data for Portuguese food retail workers and offers a practical foundation for the ergonomic redesign of retail workstations and equipment, with the potential to reduce WMSD risk and improve worker health and productivity.

## Data Availability

The original contributions presented in the study are included in the article/supplementary material, further inquiries can be directed to the corresponding authors.

## Footnotes

1
https://www.who.int/data/gho/data/themes/topics/topic-details/GHO/body-mass-index
